# The Treatment of Nævi

**Published:** 1893-10-07

**Authors:** 


### PADDINGTON GREEN CHILDREN'S HOSPITAL.

### The Treatment of Nævi.

In the casualty and surgical departments of almost every children's hospital nævi are among the more common of the minor surgical cases which present themselves for treatment. This treatment is, no doubt, in many cases simple enough, and yet a synopsis of some of the methods which have met with most success, and which are nearly always employed here, may prove useful. If, in hospital practice, parents are met with who grumble about the slight scar left after a nævus has been destroyed, not having been previously warned that a scar must of necessity remain, how much more will this be the case in private practice? Here, if anywhere, an endeavour must be made to remove them completely, and with as little resulting scar as possible. On these two points, total removal and a minimum of scar, the various methods of treatment depend—of course, guided by the nature of the nævus which has to be dealt with. For practical purposes, nævi may be classed as (1) superficial—where the skin only, or to a very slight degree the subcutaneous tissue as well, is involved—and (2) deep—where the subcutaneous tissues are involved, and the skin is either free, or involved to a greater or less extent. The first group comprises the ordinary capillary nævi; the second, the cavernous or venous nævi, complicated possibly by a capillary nævus of the skin as well.

When the skin is not involved, the diagnosis depends on the fact that the nævi can be emptied by compression, again filling up when this pressure is removed. Mothers will volunteer the information that it often gets larger when the child cries, and then becomes of a dusky colour; and that the skin over it has usually a bluish appearance. Of course, every swelling which has a nævus of the skin over it must not be mistaken for a venous nævus. Other tumours may by chance, or by some pecularity, have the skin over them the seat of a nævus; as in the case of a child who was seen here, and who had a small spina-bifida of the lower lumbar vertibræ, the skin, and to a slight extent the subcutaneous tissue, over which was the seat of an extensive capillary nævus.

*Treatment.*—In the superficial form, where the skin is alone involved, as good results as any are got from painting with the ethylate of sodium. This is done by rubbing it into the nævus with a pointed piece of wood. This rapidly causes coagulation of the blood in the capillaries, the area assuming a brownish black colour. The smarting produced by its application does not last long, and may be relieved to some extent by exposing the painted surface to the vapour of chloroform. A crust or scab forms, and when this separates some red points may remain, necessitating a fresh application. The resulting cicatrix is not deep, and does not show markedly after a few months, unless the nævus has been a very extensive one. Ethylate of sodium is much preferable to nitric acid, as with this latter, unless extreme caution is used, a nasty ulcer may be produced.

The Paquelin thermo-cautery is used in some cases where the nævus extends slightly beyond the skin, and in places where the scar is not likely to show, as the cicatrix is usually a well marked one.

If a superficial nævus shows signs of cicatricial tissue in its centre, and is not apparently growing, it is just as well left alone, as it will probably cure itself. Cure may be hastened by keeping up a certain amount of even pressure on it.

Among superficial nævi are what are known as "spider nævi." They are seen most commonly on the face as little red central points with dilated capillaries radiating from them. They are best treated by taking a large pin, and having dipped the point into some ethylate, inserting it into the central red spot. The blood here is coagulated, and usually as it disappears the radiating capillaries go as well.

Deep nævi, if so situated as to cause no disfigurement, &c., and showing no tendency to growth, may be left alone. If they show a tendency to rapid growth, probably the best mode of treatment is by excision. This, of course, must be guided by the size of the nævus, the parts contiguous to it, and also to a considerable extent as to whether the skin is involved or not. If the skin is involved and other circumstances permit—*i.e.*, given sufficient skin to form flaps, &c., excision is much the more preferable mode of treatment. The wound heals readily; its resultant scar is a mere line, and usually can be made in such a way as to produce no appreciable disfigurement. If care be taken, the nævus is also entirely removed, and no further growth will take place. If on the other hand, almost any other method is employed, the resulting scar is usually extensive, occupying the whole seat of the skin which has been involved.

The usual method of procedure here is as follows: The skin is thoroughly cleansed and shaved if this is deemed necessary, and then scrubbed with strong mixture—equal parts of 1 in 20 carbolic acid, 1 in 500 corrosive sublimate solutions. The incision is made so as to remove no more of the skin than is absolutely necessary, and the nævus is then rapidly dissected away. Bleeding is usually profuse, but is easily controlled. The bleeding points are caught up, and the vessels either twisted or tied. The skin is then brought into close apposition by a continuous suture and dressings of double cyanide gauze and salicylic wool are applied. A useful method of dressing in some cases is to lay a strip of gauze several layers in thickness along the wound. This is then covered over with a piece of some size, one layer thick and wrung quite dry. It is then coated over with collodion, which keeps the air out and fastens the dressing to the skin. A small pad of salicylic wool fixed over it helps to keep it secure. About the fifth or sixth day the stitches can be removed, and it will be quite healed in ten days.

In some cases, when it is desirable to remove them with the loss of as little blood as possible, two hare-lip pins may be passed cross-wise well beneath the nævus, and through healthy skin, and a silk ligature tied tightly beneath them. The nævus can then be removed with almost no bleeding, although of large size. The only other method employed here in the treatment of deep nævi is electrolysis. It answers well, and probably is the best way of dealing with those about the face or where a scar would disfigure, or in those cases where the skin is involved, and where suitable flaps cannot be obtained. In this case of necessity a scar must result, but then the cicatricial contraction in the case of electrolysis is not nearly so marked as in the case of the cautery, &c. It is comparatively easily carried out. At this hospital a battery is used, consisting of forty Leclanché elements so arranged that by means of switches as many cells as required can be thrown into action and also the current can be reversed at will. Attached to the one pole is a wire carrying at its distal end a platinum needle insulated to a quarter-inch from its point, and to the opposite pole a wire with three needles similarly insulated. The number of needles used is merely a matter of choice, and depends a good deal on the size of the nævus to be electrolysed. Having ascertained by the galvanometer which is the positive pole, it is arranged so that the single needle is connected with it. This is then inserted into the centre of the nævus, and the others are inserted at intervals round about, care being taken that the needles from opposite poles do not touch. Turning on the current, at first about eight cells are used, and then the number is gradually increased, the object aimed at being to solidify the nævus. The main points to guard against are the use of too many cells and the consequent risk of setting up sloughing. Usually no more than from fifteen to twenty cells are required. It is advisable before withdrawing the needles to reverse the current for a short time. In fact in large nævi it is well to do so frequently during the process of solidification. In place of having the single needle as the positive pole and inserting it into the nævus a flat plate applied to the surface of the patient's body, usually along the spine, may be used. The plate is covered with wash leather and before use is thoroughly soaked in tepid acidulated water, the patient's body being also thoroughly moistened.

